# Single-treatment tumor ablation with photodynamic liposomal irinotecan sucrosulfate

**DOI:** 10.1016/j.tranon.2022.101390

**Published:** 2022-03-12

**Authors:** Sanjana Ghosh, Boyang Sun, Dushyant Jahagirdar, Dandan Luo, Joaquin Ortega, Robert M. Straubinger, Jonathan F. Lovell

**Affiliations:** a Department of Biomedical Engineering, University at Buffalo, State University of New York, Buffalo, NY, 14260, United States of America; b Department of Chemical and Biological Engineering, University at Buffalo, State University of New York, Buffalo, New York, 14260, United States of America; c Department of Anatomy and Cell Biology, McGill University, Montreal, Quebec H3A 0C7, Canada; d CSL Behring LLC, 1020 1st Avenue, King of Prussia, PA, 19406, United States of America; e Department of Pharmaceutical Sciences, University at Buffalo, State University of New York, Buffalo, NY United States of America

**Keywords:** Irinotecan, CPT-11, Liposomes, Porphyrin-phospholipid, Chemophototherapy

## Abstract

Irinotecan (IRI) loaded actively into PEGylated liposomes via a sucrosulfate gradient has been approved recently to treat advanced pancreatic cancer. In this study, a similar liposomal composition was developed that includes a low mole fraction (1 mol.%) of porphyrin-phospholipid (PoP), a photosensitizer that stably incorporates into liposomes, to confer light-triggered IRI release. IRI-loaded PoP liposomes containing ammonium sucrosulfate (ASOS) as a complexing agent were more stable in serum compared to liposomes employing the more conventional ammonium sulfate. Without irradiation, PoP IRI liposomes released less than 5% IRI during 8 h of incubation in bovine serum at 37 °C, but released over 90% of the drug within minutes of exposure to red light (665 nm) irradiation. A single treatment with IRI-PoP liposomes and light exposure (15 mg/kg IRI with 250 J/cm^2^) resulted in tumor eradication in mice bearing either MIA PaCa-2 tumors or low-passage patient-derived tumor xenografts that recapitulate characteristics of the clinical disease. Analogous monotherapies of IRI or photodynamic therapy were ineffective in controlling tumor growth. Enhanced drug uptake could be visualized within laser-treated tumors by direct in situ imaging of irinotecan. Biodistribution analysis of IRI, its active metabolite (SN-38), and major metabolite (SN-38 G) showed that laser treatment significantly increased tumor accumulation of all IRI-derived molecular species. A pharmacokinetic model that hypothesized tumor vasculature permeabilization as the primary reason underlying the increased drug deposition accounted for the enhanced drug influx into tumors.

## Highlights

• A liposomal formulation of irinotecan that contains a non-exchangeable, embedded photosensitizer gives rise to enhanced serum stability and rapid light-triggered release when sucrosulfate is used as a trapping agent.
• Pharmacokinetic and biodistribution studies reveal enhanced delivery of Irinotecan, active metabolite SN-38 and major metabolite SN-38 following tumor irradiation with red laser light, leading to ablation of MIA-PaCa2 tumors and low-passage pancreatic cancer patient-derived xenogratfs with a single treatment.
• A pharmacokinetic model accounts for enhanced drug delivery based on photodynamic laser irradiation increasing tumor vascular permeability.

## Introduction

Liposomes have been used extensively as drug delivery systems for cancer chemotherapy [1]. From an economic and pharmaceutical technology perspective, active loading of chemotherapy drugs into the aqueous core of liposomes has been instrumental in the success of many formulations [2], [3], [4], [5]. Irinotecan (IRI; also referred to as CPT-11) is a cancer chemotherapy prodrug that inhibits topoisomerase I [6,7] and is employed in the standard-of-care pancreatic cancer treatment regimen FOLFIRINOX, that also includes oxaliplatin, fluorouracil (5-FU), and folinic acid (LV). IRI is metabolized by esterases to form the active metabolite SN-38, which is metabolized subsequently into SN-38 G by glucuronidation [8], [9], [10], [11]. Several liposomal IRI formulations have been developed [12], [13], [14], including the nanoliposome IRI formulation ONIVYDE® (nal-IRI), which was approved recently in the United States for treating metastatic pancreatic adenocarcinoma. This liposome formulation was developed with a unique remote loading technique using ammonium sucrosulfate (ASOS) as the trapping agent [14], rather than ammonium sulfate (AmSO_4_), sodium citrate or other salts which have more frequently been used [15,16]. The nal-IRI formulation showed efficacy in preclinical studies with human breast (BT474) and colon (HT29) xenograft mouse models [14], and its superior anti-tumor efficacy over free IRI has been reported in several in vivo tumor models including pancreatic, colorectal, breast, gastric, lung and cervical cancers, as well as Ewing's sarcoma tumors [14,17,18]. The improved efficacy of liposomal IRI over the free drug is thought to derive from the difference in pharmacokinetic behavior of these two drug forms, with the liposomal drug having a longer circulation time. Phase I clinical trials of liposomal IRI as monotherapy for advanced solid tumors showed that the maximum tolerated dose is 120 mg/m^2^ at 3-weeks interval [19]. Phase II clinical trials showed improved response rates for ONIVYDE in esophagogastric cancer patients [20], unresectable metastatic colorectal cancer response rates [21], and for metastatic pancreatic cancer [22]. The Phase III randomized open-label NAPOLI-1 clinical trial reported improved therapeutic efficacy of ONIVYDE® combined with fluorouracil and folinic acid (5-FU/LV) compared to either ONIVYDE® alone or 5-FU/LV alone, in patients with advanced metastatic pancreatic ductal adenocarcinoma following gemcitabine-based therapy [23]. Despite this clinical success, the trial showed that ONIVYDE® extended median survival by less than two months compared to either monotherapy arms, leaving room for further improvement with alternative approaches. In the present study we develop such an approach that makes use of a liposomal formulation similar to ONIVYDE®, however modifies it with the capability of on-demand temporal and spatial control over drug release. The overall rationale is to demonstrate the feasibility of developing such a formulation and demonstrate its efficacy in using light-irradiation to better deliver IRI and SN-38 to tumors for ablative responses.

The emerging field of chemophototherapy (CPT) combines photodynamic therapy (PDT) with chemotherapy, and can potentiate the local efficacy of cancer treatment by providing temporally- and spatially-controlled drug delivery to tumors [24]. Liposomes represent an excellent test-case for CPT development as they have been used to deliver photosensitizers in preclinical and clinical pharmaceutical formulations [25]. Porphyrin-phospholipid (PoP) is a unique photosensitizer that consists of a porphyrin conjugated to a phospholipid and has the advantages of straightforward and stable incorporation into liposome bilayers. Inclusion of PoP in the liposome membrane also allows for stimulus-dependent release of cargo with red light illumination [26]. An additional advantage of PoP is that PDT-mediated damage to the tumor vasculature increases the uptake of drugs in tumors [27,28]. Motivated by the success of ONIVYDE®, we sought to develop IRI-PoP liposomes to provide light-mediated control of IRI release, and to test the hypothesis that a triggered-release formulation that better delivers SN-38 to tumors could be effective for chemophototherapy in human pancreatic cancer mouse models.

## Materials and methods

### Liposome preparation, drug loading and characterization

Lipids were acquired from Corden Pharma International, and other materials were acquired from Sigma unless mentioned otherwise. Porphyrin phospholipid (PoP) was synthesized as described [26]. 1,2-distearoyl-sn‑glycero-3-phosphocholine (DSPC, Corden Pharma catalog # LP-R4–076), cholesterol (PhytoChol, Wilshire Technologies Inc. catalog # 57–88–5) and 1,2- distearoyl-sn‑glycero-3-phosphoethanolamine-N-[methoxy(polyethylene glycol)−2000] (MPEG-2000-DSPE, Corden Pharma catalog # LP-R4–039) and IRI (LC Laboratories catalog # I-4122) were used. A method based upon [14] was developed to load PoP liposomes with IRI. For a 5 mL batch (20 mg/mL lipids), liposomes were prepared by injecting 1 mL ethanol at 60 °C into powdered lipids (DSPC: Chol: PoP: MPEG-2K-DSPE in the molar ratio of 58.7:40:1:0.3), followed by rapid addition of 4 mL of pre-warmed 120 mM ASOS (Toronto Research Chemicals, catalog # S698990) at 60 °C. The mixture was then passed 10 times through a nitrogen-pressurized extruder (Northern Lipids) having sequentially stacked polycarbonate membranes of 0.2, 0.1 and 0.08 µm pore size. To remove free ASOS and ethanol, liposomes were dialyzed against 800 mL of 145 mM sodium chloride with 5 mM HEPES (pH 6.5) with at least two changes of buffer. IRI was loaded in the liposomes by adding the drug (IRI dissolved in water at 10 mg/mL IRI) at a drug:lipid molar ratio of 1:8 for 1 hr at 60 °C. Unencapsulated IRI was removed using a Sephadex G-75 column. IRI was quantified by fluorescence on a TECAN Safire fluorescent microplate reader using an excitation wavelength of 370 nm and an emission wavelength of 435 nm. IRI encapsulation efficiency was quantified as the percentage of drug fluorescence that co-eluted with the liposome fraction (by lysing the liposomes with 0.5% Triton X-100) from a Sephadex G-75 column equilibrated with PBS running buffer. Liposome size and polydispersity index (PDI) were measured in PBS using a NanoBrook 90 instrument with phase analysis dynamic light scattering.

The serum stability of the IRI liposome formulations was studied by diluting the liposomes (2 mg/mL IRI) 200 fold in 20% bovine serum at 37 °C while recording %IRI released due to leakage at different time points using fluorescence measurements over 24 h using a microplate reader as described above. The percentage IRI release was determined by using the formula:%Release = (F_final_-F_initial_) /(F_(TX-100)_-F_initial_) × 100%. The initial fluorescence of IRI liposomes was read at 0 hr. The final fluorescence values were read at different time points: 0.1 h, 0.5 h, 1 h, 2 h, 4 h, 8 h and 24 h. F_(TX-100)_ is the fluorescence measured after adding 0.5% Triton X-100 to lyse the liposomes, representing maximum fluorescence of the drug encapsulated inside liposomes.

### Cryo-TEM

Sample vitrification was performed using a Vitrobot Mark IV (Thermo Fisher Scientific). Holey carbon grids (C-Flat 2/2–2Cu-T) were washed with chloroform for 2 h before sample vitrification. Grids were treated with negative glow discharge in air at 5 mA for 15 s before the sample was applied. To apply the sample on the grid, before vitrification a volume of 3.6 μL of sample was first applied to the grid and manually blotted using Vitrobot blotting paper (Standard Vitrobot Filter Paper, Ø55/20 mm, Grade 595). We then did a second application of the sample on the grid using a volume of 3.6 μL of sample that was applied to the grid. We then blotted once with Vitrobot paper for 3 s with a blot force +1 before the grid was plunged into liquid ethane. The Vitrobot was set at 25 °C and 100% relative humidity. Grids were loaded into a Tecnai F20 electron microscope operated at 200 kV using a Gatan 626 single tilt cryo-holder. Data acquisition was performed using Serial-EM software using a FEI Tecnai G2 F20 microscope at 200 kV equipped with a Gatan Ultrascan 4000 4k x 4k CCD Model 895 Camera System. All images were collected at a magnification of 62,000x, which produced images with a calibrated pixel size of 1.830 Å. Images were collected with a total dose of ∼ 50 *e*^−^/Å^2^ using a defocus ranging from − 2.25 μm to − 2.75 μm.

### Light-triggered release of PoP liposomes

IRI-PoP liposomes (2 mg/mL IRI) were diluted 1000-fold in 50% bovine serum (Pel-Freeze catalog # 37,225–5) and irradiated at 37 °C with a 665 nm diode laser (RPMC laser, LDX-3115–665) at a fluence rate of ∼300 mW/cm^2^. A custom PTI fluorometer setup enabled simultaneous irradiation and monitoring of IRI fluorescence, which increases during the course of release from liposomes. Drug release was calculated in real time using the equation:%Release = (F_final_-F_initial_) /(F_TX-100_-F_initial_) × 100%.

### Liposome storage stability

Three separate batches of IRI-PoP liposomes, prepared with an initial drug:lipid molar ratio of 1:8, were stored in closed amber vials at 4 °C, and drug retention, particle size and polydispersity, serum stability, and light-triggered release rates were assessed every two weeks for 3 months. Liposome sizes and polydispersity were measured by dynamic light scattering in PBS. Serum stability was measured as described above by measuring%IRI released after 6 h of incubation at 37 °C.

### Tumor growth inhibition studies

All animal study guidelines were followed, based on protocols approved by the Institutional Animal Care and Use Committees (IACUC) at the University at Buffalo and Roswell Park Comprehensive Cancer Centers. Two tumor model systems were employed to evaluate the efficacy of IRI-PoP liposomes. For the MIA PaCa-2 xenograft model, 5 × 10^6^ MIA PaCa-2 cells were injected subcutaneously in the right flank of five week old female nude mice. For the PaCa patient-derived-xenograft (PDX) tumor #14,312, which was developed at the Roswell Park Comprehensive Cancer [29], tumors were carried in male SCID mice. For implantation, donors were euthanized under anesthesia, and tumors were harvested rapidly and immersed in ice-cold culture medium. Tumor fragments of 2 × 2 × 2 mm were cut under ice-cold medium and implanted subcutaneously into anesthetized mice through a small incision in the lateral abdominal wall. The incision was closed with a staple. When tumors reached 4 to 6 mm in diameter, mice were randomized and grouped for treatment. The intravenous dose for IRI-PoP liposomes was 15 mg/kg of liposome-encapsulated IRI. The dose of PoP in empty PoP liposomes was equivalent to the PoP dose administered as IRI-PoP liposomes. Laser treatment groups were irradiated 1 hr after drug administration using a 665 nm laser (RPMC laser, LDX-3115–665) at a fluence rate of 200 mW/cm^2^, for a total fluence of 250 J/cm^2^, unless indicated otherwise. Only a single treatment was used. Tumor size was monitored 2–3 times per week using calipers, and tumor volumes were calculated with the ellipsoid formula: Volume = π × length × width × height / 6. Mice were sacrificed when the tumor exceeded 1.5 cm in size or if the tumor ulcerated (for PDX tumors).

### Biodistribution and pharmacokinetics of IRI-PoP liposomes in tumor and other tissues

For PK analysis, five-week old female nude mice were inoculated subcutaneously on both flanks with 5 × 10^6^ MIA PaCa-2 cells. When the tumors reached 5 - 6 mm in diameter, the mice were injected intravenously with IRI-PoP liposomes at a dose of 15 mg/kg IRI. Tumors on the right flank of each mouse were treated with a 665 nm laser as described above, with a drug-light interval of 1 hr. The opposing tumor was not irradiated. The mice were sacrificed 1, 2, 8, 24, 48 or 96 h after drug administration, and the tumors from each animal, along with the spleen, liver, and plasma were harvested rapidly after euthanasia. Drug was extracted by homogenizing the tissues in ice cold 20% methanol in water at a final concentration of ∼200 mg/mL tissue. Blood was collected in blood collection tubes containing EDTA as anticoagulant. The plasma was collected by centrifuging the blood at 2000 × g for 15 min. The tissues and plasma were stored at −80 °C. For analysis, the samples were allowed to come to room temperature. PoP concentrations were determined by fluorescence measurements based upon a standard curve using a method described previously [30]. Mouse plasma and tissues samples were analyzed for IRI and its metabolites, SN-38 and SN-38 Glucuronide (SN-38 G), using a high-pressure liquid chromatographic assay with tandem mass spectrometric detection (LC-MS/MS) over a calibration range of 0.200 – 200 ng/mL for CPT-11, SN-38 and SN-38 G. CPT-11, SN-38, and SN-38 G (Toronto Research Chemicals, Toronto, Canada) were used to prepare stock solutions to a concentration of 1.00 mg/mL in dimethyl sulfoxide. The solutions were wrapped in foil to protect from light and stored at −20 °C. CPT-11-d_10_ (Toronto Research Chemicals, Toronto, Canada) and camptothecin (Sigma-Aldrich, St. Louis, Missouri) were utilized as internal standards (IS) and prepared in a similar fashion with CPT-11-d_10_ dissolved in methanol and camptothecin dissolved in a 4:1 chloroform: methanol mixture.

All sample manipulations were performed under yellow lights and on ice. Following the addition of 100 µL of homogenized tissue or plasma samples, or plasma calibration and quality control samples to a microcentrifuge tube, protein precipitation was performed using ice-cold acidified methanol (0.1% acetic acid). Samples were centrifuged at 13,500 rpm for 10 min at 4 °C and a portion of the supernatant injected for analysis. LC-MS/MS analysis of the extracted samples was performed using a Shimadzu Prominence (Kyoto, Japan) high pressure liquid chromatography (HPLC) system and a Sciex 5500 mass spectrometer (Sciex, Framingham, Massachusetts) with electrospray ionization source (ESI).). The analytes were chromatographed over a CORTECS™ C18+ HPLC column (100 × 2.1 mm, 2.7 µm, part number 186,007,397, Waters, Milford, MA) preceded by a CORTECS™ C18+ VanGuard cartridge (2.1 × 5 mm, 2.7 µm, part number 186,007,685, Waters) using a biphasic gradient. Briefly, initial conditions of mobile phase A: mobile phase B / 90%:10% was held for 0.3 min, then B is increased to 100% over 4 min. 100% B was held for 2 min then brought back to initial conditions (0.5 min) and allowed to re-equilibrate for 2.5 min Mobile phase A consisted of 0.1% acetic acid in water while mobile phase B consisted of 0.1% acetic acid in acetonitrile, and the column was maintained at 50 °C. The analytes were detected using multiple reaction monitoring (MRM) in positive ion mode. Parent/fragment ion pair transitions were 587.400 → 195.100 *m/z* for CPT-11, 393.200 → 249.200 *m/z* for SN-38, 569.400 → 349.200 *m/z* for SN-38 G, 596.900 → 177.200 *m/z* for CPT-11-d_10_ (IS used to quantitate CPT-11 and SN-38 G), and 349.100→ 219.100 *m/z* camptothecin (IS used to quantitate SN-38). Retention times were 3.34 min for CPT-11 and CPT-11-d_10_, 3.46 min for SN-38 G, 3.87 min for SN-38 and 4.00 min for camptothecin. All sample results were obtained within four analytical runs where the assay performance is shown in **Table SI** and **TableS2**. Each analytical run contained calibration samples at the following concentrations: 0.200, 0.250, 0.500, 1.00, 5.00, 10.0, 50.0, 100 and 200 ng/mL, with quality control samples at 0.750, 7.50, and 75.0 ng/mL of each analyte. If the initial result for a sample was above the upper limit of quantitation (200 ng/mL), the analysis was repeated with an appropriate dilution factor to obtain a result within the calibration range. While the LC-MS/MS method was not validated, FDA acceptance criteria (Guidance for Industry: Bioanalytical Method Validation, U.S. Department of Health and Human Services, Food and Drug Administration, May 2001) were applied to the calibration and quality control samples; specifically, a calibration curve must have a minimum of 6 passing calibrators with individual and mean back-calculated concentrations for each calibrator and quality control sample having accuracy results equal to or within the range of 100±15% of the nominal value, or 100±20% at the lower limit of quantitation (LLOQ). Calibrator and quality control samples must also have precision (percent relative standard deviation;%RSD) of ≤15%, with ≤20% being allowed at the LLOQ.

*Pharmacokinetic data analysis:* ADAPT 5 software [31] was used for data fitting and simulation, and the maximum likelihood method was used. The replicate data at each time point in each experiment were naïve-pooled. The IRI and SN-38 tumor PK for both tumors, with or without laser treatment, were fitted sequentially, as were the IRI and SN-38 plasma PK. The goodness of fit and model selection was assessed by visual inspection of fitted curves, objective function values such as Akaike information criterion (AIC), improved likelihood, and precision (CV%) of the estimated parameters. The variance model for model fitting was:

$$V_{i}=V\left(\theta ,\sigma ,t\right)=[{\left(\sigma 1+\sigma 2\cdot Y\left(\theta ,t_{i}\right)\right]}^{2}$$

where V(θ,σ,t) is the variance for the *ith* point, $Y(\theta ,t_{i})$ is the *ith* model predicted value, θ represents the estimated structural parameters, and σ1 and σ2 are the variance parameters that were estimated. Non-compartmental PK analysis was performed using MS Excel from MS Office 2019 with the log-linear trapezoidal method.

A mono-exponential model was used to describe the pharmacokinetic profile of IRI and SN-38 in plasma and tumor tissue. The equations and initial conditions of the PK model are described below. The PK of IRI and SN-38 in plasma are represented by the following equations:

$$Vp\frac{dCp}{dt}=\;-CL*Cp,\;IC=Dose$$

$$Vp\frac{dCp^{\prime }}{dt}=\;CLm*Cp-CL^{\prime }*Cp^{\prime },\;IC=0$$

where Cp and Cp’ represent the concentrations of IRI and SN-38, respectively, in plasma. CL and CL’ respectively represent the clearance of IRI and SN-38 from the central plasma compartment. CLm (the clearance of IRI into its metabolite SN-38) is a part of CL parameter, where CL=CL_m_+CL_others_). The PK of IRI and SN-38 in the tumor compartment are described by the following equations:

$$\frac{dCt}{dt}=\;Kin*Cp-Kout*Ct,\;IC=0$$

$$\frac{dCt^{\prime }}{dt}=\;Kin^{\prime }*Cp^{\prime }-Kout^{\prime }*Ct^{\prime },\;IC=0$$

The PK of IRI and SN-38 in the tumor compartment after laser treatment are represented by the following equations:

$$\frac{dCt}{dt}=b1*Kin*Cp-b2*Kout*Ct,\;IC=0$$

$$\frac{dCt^{\prime }}{dt}=b1^{\prime }*Kin^{\prime }*Cp^{\prime }-b2^{\prime }*Kout^{\prime }*Ct^{\prime },\;IC=0$$

where Ct and Ct’ represent the tumor concentration of IRI and SN-38 respectively, Kin is the influx rate constant of IRI into tumor without laser, Kout is the efflux rate constant of IRI from tumor without laser, b1 is the enhanced permeabilization factor on the influx rate of IRI due to laser treatment, b2 is the enhanced permeabilization factor that modifies the tumor efflux rate of IRI due to laser treatment, Kin’ is the influx rate constant of SN-38 into tumor without laser, Kout’ is the efflux rate constant of SN-38 without laser, b1’ is the enhanced permeabilization factor on the influx rate of SN-38 due to laser treatment, and b2’ is the enhanced permeabilization factor on the tumor efflux rate of SN-38 due to laser treatment.

### Tumor microdistribution of IRI and PoP

As described for therapeutic experiments, mice bearing MIA PaCa-2 or PDX tumors were injected intravenously with IRI-PoP liposomes at an IRI concentration of 15 mg/kg. Tumors were irradiated 1 hr post-injection with a 665 nm laser at a laser fluence rate of 200 mW/cm^2^ and a total fluence of 250 J/cm^2^. The mice were sacrificed 8 hr after drug administration and the tumors were harvested rapidly and immediately flash-frozen in liquid nitrogen after embedding tissue in OCT mounting medium. Frozen sections (8 µm) were prepared using a Cryostat (H/I Bright OTF5000) and stored at −20 °C. The tumors were imaged with a fluorescence microscope (EVOS FL Auto) using a DAPI filter cube (357 nm excitation; 477 nm emission) for IRI and a custom filter cube (400 nm excitation; 679 nm emission) for PoP.

### Statistical analysis

Statistical analysis of data was performed using GraphPad Prism software Version 6.01 using methods indicated in the captions.

## Results and discussion

**Fig. 1:**
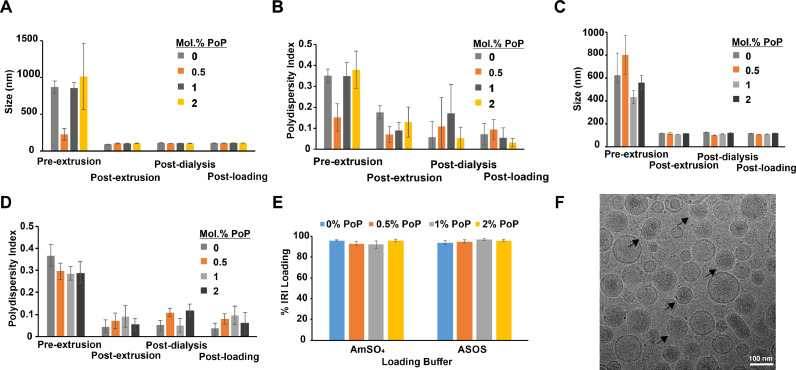
**Formation of PoP liposomes and loading with IRI.** IRI was loaded in liposomes containing varying amounts of PoP and two different complexing agents (ASOS or AmSO_4_). **A)** Size and **B)** polydispersity index of AmSO_4_-containing liposomes; **C)** size and **D)** polydispersity index of ASOS-containing liposomes. **E)** Liposomal loading efficiency of IRI. Values represent mean ± std. dev. for *n* = 3 preparations. **F)** Cryo-TEM image of 1 mol.% PoP IRI-loaded liposomes with ASOS. Arrows point out that most liposomes are unilamellar with an electron dense (darker) aqueous core, due to drug loading. Scale bar, 100 nm.

IRI was actively loaded into liposomes composed of DSPC:Chol:MPEG-2K-DSPE (58.7:40:0.3), which is similar to the lipid composition of ONIVYDE [14]. PoP was included in the liposomes at ratios of 0.5, 1.0, or 2.0 mol.%, by substituting DSPC with an equivalent mol.% of PoP. Two different active loading buffers were employed that compared AmSO_4_ and ASOS as IRI complexing agents. The size and polydispersity index (PDI) of the liposomes decreased with extrusion, regardless of the complexing agent (Fig 1**A-**1**D**). All formulations showed greater than 90% loading efficiency (Fig 1**E**), and liposome size changed minimally after IRI loading. The PDI of the drug-loaded liposomes was less than 0.1, reflecting a monodisperse particle population. The morphology of ASOS IRI-PoP liposomes containing 1 mol.% PoP, as assessed by cryo-transmission electron microscopy, (Fig 1**F)**. Spherical, unilamellar liposomes were apparent, with an electron dense interior corresponding to the actively-loaded IRI. These results show that PoP can be incorporated into small unilamellar liposomes employing either AmSO_4_ or ASOS gradients for active loading of IRI.

**Fig. 2:**
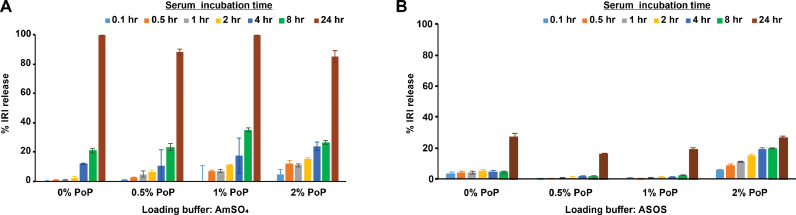
**Effect of drug-complexing agent on in vitro serum stability of IRI loaded PoP liposomes.** IRI was loaded into liposomes containing the indicated PoP mole fraction with different remote loading buffers. In vitro serum stability was tested by incubation in 20% bovine serum at 37 °C. **A)** Serum stability of IRI-PoP liposomes using AmSO_4_ as the remote loading buffer **B)** Serum stability of IRI-PoP liposomes using ASOS as the remote loading buffer. Mean ± std. dev. for *n* = 3.

To determine which complexing agent (ASOS or AmSO_4_) affords greater serum stability in liposomes that would enable longer blood circulation time in vivo, the serum stability of IRI-loaded PoP liposomes was assessed. Fig 2**A** shows that the drug efflux rate from liposomes employing AmSO_4_ was greater than from ASOS-based formulations; AmSO_4_-containing IRI-PoP liposomes incubated in 20% serum at 37 °C released 20% of the encapsulated IRI within 8 h, and essentially all the drug within 24 h. In general, liposomes containing a higher PoP mol% were less stable, likely due to disruption to bilayer packing by the bulky PoP moiety. As shown in Fig 2**B**, IRI-PoP liposomes loaded using ASOS with 0–1.0%mol PoP had markedly improved serum stability, releasing less than 5% of the drug over 8 h of incubation. **Fig S1** shows the difference in IRI release at different time points in 1% PoP liposomes loaded via ASOS or AmSO_4._ ASOS-containing IRI-PoP liposomes having 2 mol.% PoP were somewhat less stable and released IRI more rapidly over 8 hr, but overall, IRI-PoP liposomes employing ASOS were more stable in serum than those employing AmSO_4_ as a drug-complexing agent. How ASOS provides better serum stability to this formulation is of interest but was not assessed further. Presumably, the sucrosulfate-IRI aggregates trapped in the internal liposomal aqueous space are more stable than the analogous sulfate-IRI aggregates. Given the superior stability of liposomes employing ASOS as a complexing agent, and their likeness to clinically-validated ONIVYDE®, this formulation was selected for further studies.

**Fig. 3:**
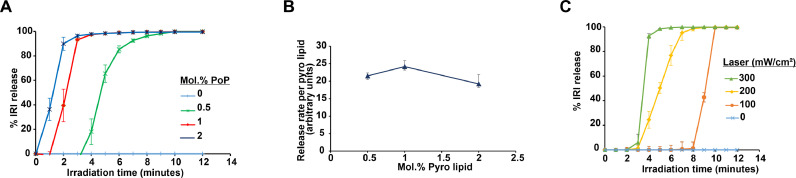
**Light-triggered release from IRI loaded PoP liposomes.** IRI was loaded into PoP liposomes using ASOS as a complexing agent, and light-triggered release was assessed at 37 °C in 20% bovine serum. Liposome samples were diluted 1000-fold and irradiated using a 665 nm diode laser. **A)** Time course of IRI release from IRI-PoP liposomes containing varying mole fractions of PoP at a laser fluence rate of 300 mW/cm^2^. **B)** IRI release rate normalized by the PoP content of the liposomes. **C)** IRI release from 1 mol% PoP liposomes at different laser fluence rates. Mean ± std. dev. for *n* = 3.

Light-triggered release of IRI from PoP liposomes was investigated for formulations loaded with ASOS as the complexing agent and containing varying amounts of PoP in the bilayer. We previously found that 2 mol.% PoP liposomes in a Doxil-like formulation enabled optimal light-triggered release of doxorubicin when irradiated with near-infrared light [30]. Fig 3**A** shows triggered release from IRI-PoP liposomes in 20% bovine serum at 37 °C when irradiated with a 665 nm diode laser at a fluence rate of ∼300 mW/cm^2^ for 15 min. IRI release increased with increasing PoP content, and all formulations released more than 90% of the drug in less than 6 min. In contrast, no drug was released from light-irradiated IRI liposomes lacking PoP. This indicates the requisite role played by PoP in drug light-triggered release. Fig 3**B** shows the IRI release rate normalized by the mole fraction of PoP present in the bilayer. The normalized release rate of 1% IRI-PoP liposomes was slightly higher than the other formulations. Generally, the IRI release rate was faster than observed previously for doxorubicin release, possibly reflecting a higher stability of the doxorubicin aggregates in the liposome core. Overall, the lower requirement for PoP to trigger drug release from IRI-PoP liposomes would provide greater serum stability, so the 1 mol.% PoP fraction was used for all further studies.

The effect of fluence rate upon light-triggered IRI release was assessed for 1 mol.% PoP-IRI liposomes in 20% bovine serum at 37 °C (Fig 3**C**). IRI release from IRI-PoP liposomes showed fluence-dependence: more than 90% of the drug was released within 10 min at a laser fluence rate of 100 mW/cm^2^, whereas with 300 mW/cm^2^, more than 90% of the drug was released in less than 4 min. No drug released was observed from the IRI-PoP liposomes without laser treatment.

**Fig. 4:**
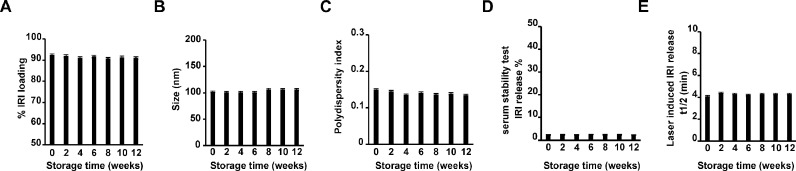
**Storage stability of IRI-loaded PoP liposomes. IRI-PoP** liposomes were prepared and stored in the dark at 4 ${}^{\circ }$C and assessed periodically for: **A)**% IRI loading **B)** Size (nm), **C)** PDI, **D)** erum stability, and **E)** Laser-induced IRI release. Data show mean ± std. dev. for *n* = 3 separately prepared batches of liposomes.

The storage stability of drug encapsulation and release rate under irradiation was assessed for 1 mol% IRI-PoP liposomes. For IRI-PoP liposomes stored in amber vials in the dark at 4 °C, more than 90% of the IRI content remained stably loaded inside the liposomes over the 12-week storage period (Fig 4**A**), with no appreciable increase in drug leakage. Fig 4**B** shows that the liposomes maintained their hydrodynamic diameter of approx. 100 nm, as well as a PDI < 0.15 (Fig 4**C**), which represents a monodisperse liposome population**.** Fig 4**D** shows the serum stability of IRI-PoP liposomes during refrigerated storage. At any time over 12 weeks, samples stored in 4 °C were taken out and incubated in 20% bovine serum at 37 °C. These liposomes in serum released less than 3% of the encapsulated IRI over a 6 hr incubation period. The light irradiation responsiveness of IRI-PoP liposomes also was evaluated during storage. Fig 4**E** shows the time required to release 50% of the drug from liposome samples when irradiated at room temperature with a 665 nm laser with laser fluence rate of 300 mW/cm^2^ for 10 min for total fluence of 180 J/cm^2^. At all sampling times over 12 weeks, IRI-PoP liposomes released > 50% of their drug content in ∼ 4 min. Thus, IRI-PoP liposomes show excellent refrigerated storage stability for at least 12 weeks, while maintaining their size, dispersity, serum stability, and their photosensitivity for rapid release of contents upon irradiation

**Fig. 5:**
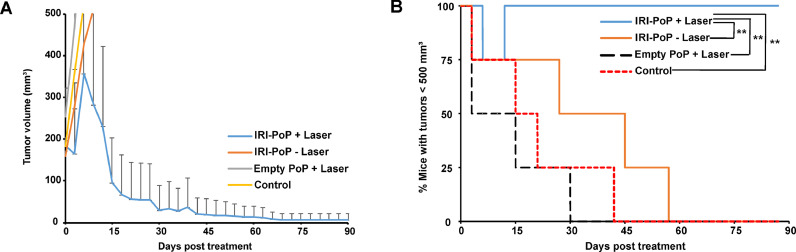
**Anti- MIA-PaCa-2 tumor efficacy with a single treatment of IRI-PoP liposomes.** Female athymic nude mice bearing subcutaneous tumors were injected intravenously with 15 mg/kg IRI in IRI-PoP liposomes or with an equivalent dose of PoP in drug-free liposomes. 1 hr following injection, mice in the +laser groups were treated with a 665 nm laser at a fluence rate of 200 mW/cm^2^ (total 250 J/cm^2^). Mice were sacrificed when tumors grew to 1.5 cm. **A)** Tumor growth curve. Each data point shows mean ± std. dev. For *n* = 4 mice per group. **B)** Percentage of mice with tumors < 500 mm^3^ for the indicated treatments. Statistical analysis was performed by Log-rank (Mantel-Cox) test, *P < 0.05, **P < 0.01. Each data point shows mean ± std. dev. for *n* = 4 mice per group.

The anti-tumor efficacy of IRI-PoP liposomes was investigated in female athymic nude mice bearing MIA PaCa-2 human pancreatic cancer cell xenografts, and treatment was initiated when tumors were ∼150–200 mm^3^ in diameter. Mice received a single intravenous dose of 15 mg/kg IRI as IRI-PoP liposomes or an equivalent dose of PoP in drug-free liposomes, and 1 h after administration of liposomes, tumors were treated with a 665 nm laser at a fluence rate of 200 mW/cm^2^ (total fluence of 250 J/cm^2^). Mice that were treated with IRI-PoP liposomes and laser-treated had complete tumor regression, and survived without any sign of tumor regrowth for 90 days (Fig 5**A**). In contrast, treatment with IRI-PoP liposomes alone (without laser) or drug-free PoP liposomes with laser showed only a modest inhibition of tumor progression, and all mice from those groups developed tumors reaching volume ∼1200 mm^3^ by day 65 (Fig 5**B**). However, it is unclear why mice that received empty-PoP liposomes with irradiation did not show faster tumor shrinkage as compared to untreated control mice.

**Fig. 6:**
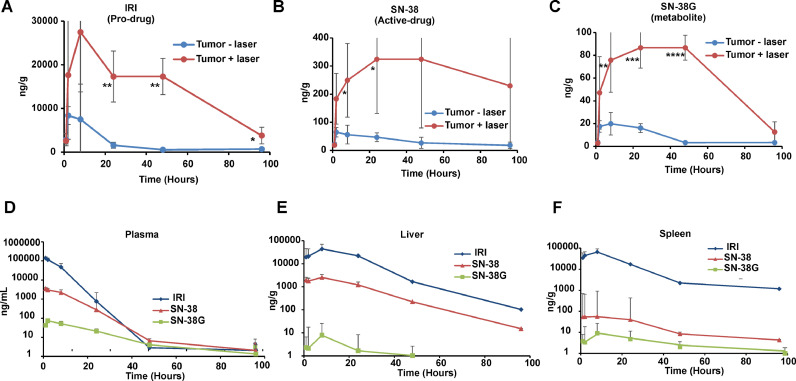
**Biodistribution kinetics of IRI-PoP liposomes in a contralateral dual tumor model.** Mice bearing contralateral MIA-PaCa-2 xenografts were administered 15 mg/kg IRI-PoP IV and 1 hr later, only one of the tumors was irradiated with a 665 nm laser at a laser fluence rate of 200 mW/cm^2^ (total 250 J/cm^2^). Accumulation of IRI, SN-38 and SN-38 G in tumors and other tissues was assessed at different time points after drug administration using an LC-MS/MS method (**Table S3**). Tumor uptake with or without laser treatment of **A)** IRI **B)** SN-38 and **C)** SN-38 G. Concentrations of IRI, SN-38, and SN-38 G in **D)** Plasma, **E)** Liver, and **F)** Spleen. Statistical analysis of tumoral drug uptake between indicated groups at different timepoints was performed by T-test, *P < 0.05, **P < 0.01, ***P < 0.001, ****P <0.0001. Data show mean ± std. dev. for *n* = 4 mice per group.

To assess mechanisms underlying the striking anti-tumor efficacy of IRI-PoP liposomes after laser treatment, the biodistribution and pharmacokinetics of IRI, active metabolite SN-38, and inactive metabolite SN-38 G were investigated in female athymic nude mice bearing dual MIA PaCa-2 tumors. Using the same treatment parameters as for the survival study, IRI-PoP liposomes were administered intravenously, and one of the two contralateral tumors was irradiated. Groups of mice were sacrificed at various time points and plasma, select organs, and both tumors were collected for drug biodistribution analysis using an LC-MS/MS method (**Table S3**). Laser irradiation mediated a drastic enhancement in tumor delivery of IRI (Fig 6**A**), SN-38 (Fig 6**B)** and SN-38 G (**6C**) in the laser-treated tumor. Tumor concentrations of IRI were substantially higher than those of the two metabolites, and reached a maximum of 8 hr after drug administration (i.e., 7 h after laser treatment). At that time, the amount of IRI in non-irradiated tumors was approx. 7000 ng/g, and was ∼4-fold greater in the irradiated tumors. For SN-38, tumor accumulation was maximal 24 hr after drug administration (23 hr post irradiation). At that time, the active metabolite SN-38 was 46 ng/g in non-irradiated tumors, and 325 ng/g in irradiated tumors, an increase of > 7-fold. For SN-38 G, the tumor deposition 8 hr post drug administration was ∼20 ng/g in tumors that were not irradiated and ∼75 ng/g in tumors that were. These values were similar at the 24 hr time point. Taken together, these data show that tumor irradiation led to a striking and sustained increase in the concentration of IRI and its major metabolites.

Concentrations of IRI, SN-38, and SN-38 G were also quantified for plasma, and two reticuloendothelial system (RES) organs, liver, and spleen (Fig 6**D**). The pharmacokinetic behavior of the parent compound and metabolites were similar in plasma; the blood circulating half-life of IRI was 5.5 hr, with an area under the plasma concentration-time curve (AUC) of 1042.85 µg(ml*hr). For SN-38, the circulating half-life was 8 hr with an AUC of 45.98 µg(ml*hr). Fig 6**E** shows the biodistribution of IRI and related metabolites in the liver at various time points. 8 hr after drug administration, the amount of IRI in the liver was about 44,000 ng/g, the amount of SN-38 was 2500 ng/g and the amount of SN-38 G was about 8 ng/g. Spleen distribution is shown in Fig 6**F.** 8 hr after drug injection, the amount of IRI was about 65,000 ng/g in the spleen, whereas the amount of SN-38 and SN-38 G in the spleen was about 55 ng/g and 9 ng/g respectively. The similar nature of distribution of the drugs in key organs implies the stability of liposomes during circulation and clearance over time. At most time points, the concentration of the drug in liver and spleen, which are organs known to eliminate liposomes from circulation was roughly only double that of the irradiated tumors.

**Fig. 7:**
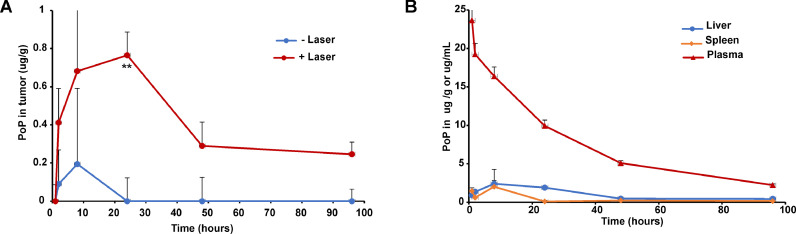
**Biodistribution of PoP via IRI loaded PoP liposomes.** Mice bearing contralateral MIA-PaCa-2 xenografts were administered 15 mg/kg IRI-PoP IV and 1 hr later, one of the tumors was irradiated with a 665 nm laser at a laser fluence rate of 200 mW/cm^2^ (total 250 J/cm^2^). Accumulation of PoP in tumor and other tissues was quantified at different time points. **A)** Tumor uptake of PoP with and without laser treatment **B)** Observed PoP concentrations in plasma, spleen and liver over time in mice treated with laser. Data show mean ± std. dev. for *n* = 4 mice at each time point. Statistical analysis of tumoral drug uptake was performed by T-test, *P < 0.05, **P < 0.01.

In addition to IRI, the accumulation of PoP in tumors and other tissues was quantified by fluorescence. Fig 7**A** shows the effect of laser irradiation on tumor accumulation of PoP. Appreciably higher PoP accumulation was observed in tumors that received irradiation. Because PoP is part of the liposome carrier and is not released during laser irradiation, this supports the mechanistic feature of photodynamic-induced vascular damage being responsible for greater tumor drug influx over time due to intravascular drug release. Fig 7**B** shows the kinetics of PoP deposition in plasma, spleen and liver over time. Overall, similar trends in the distribution of IRI and PoP in key non-tumor tissues was observed over time.

**Fig. 8:**
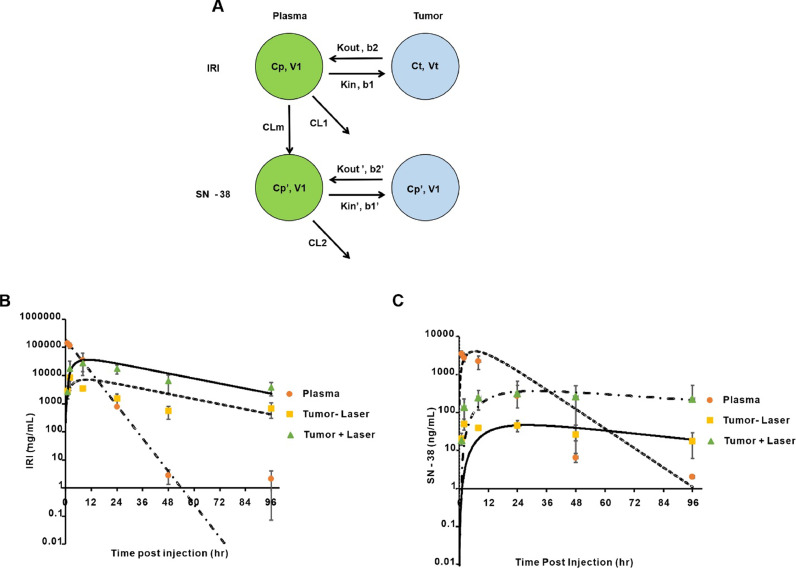
**Pharmacokinetics of IRI-PoP liposomes.** Mice were injected intravenously with IRI-PoP liposomes at a dose of 15 mg/kg IRI and then laser irradiated with a total fluence of 250 J/cm^2^ using a 665 nm laser with fluence rate of 200 mW/cm^2^ 1 hr after drug administration. The concentrations of IRI (pro-drug) and SN-38 (active drug) in plasma and tumor (laser treated/untreated) were measured. **A)** schematic of the PK model for IRI and SN-38 in plasma and tumor. Cp is the concentration of IRI in plasma. Ct is the concentration of IRI in plasma. K_in_ and K_out_ represent the influx and efflux rate of IRI in the tumor without laser treatment. K_in_’ and K_out_’ represent the influx and efflux rate of SN-38 in the tumor without laser treatment. CLm represents the clearance of IRI by conversion to SN-38 and CL = CL_m_ + CL_others._**B)** Plasma and tumor concentrations of IRI; lines represent model fits, and symbols represent the observed mean data. **C)** Plasma and tumor PK of SN-38. Data represent mean ± std. dev., *n* = 3 mice/point.

To probe mechanistic contributions underlying the enhanced tumor drug delivery following irradiation, a model IRI and SN-38 PK was developed and implemented in ADAPT 5. The model used to describe the pharmacokinetic profile of IRI and SN-38 in plasma and tumor tissue is shown in Fig 8**A** and reasonably described the observed plasma and tumor concentrations of IRI (Fig 8**B**) and SN-38 (Fig 8**C**). All parameters are well-estimated (Table 1), and the plasma PK of PoP is shown in **Fig S2**. The PK of liposomal IRI in plasma exhibited a mono-exponential model as per observation. One limitation with this model is that the plasma concentration of IRI at 96 hr was higher than the predicted concentration. The possible reason for this could be related to the long duration of time post injection of drug which has led to gradual diffusion of drug from tissues into plasma. The residual IRI from tissue or organs would have released into plasma, resulting in high IRI concentrations in plasma at 96 hr. Non-compartmental analysis showed that the PK of liposomal IRI had a relatively fast clearance, with a circulating half-life of 5.5 hr and a clearance of 0.36 mL/hr (Table 2). However, considering the limited data obtained in the study, a less complicated model was feasible. Nonetheless, the simplified PK model still describes the experimental data reasonably well.

**Table 1:** Fitted PK parameters of IRI model.

**Parameters**	**Definition**	**Final Estimate**	**Units**	**CV%**
V_p_	Volume of central plasma compartment	1.95	mL	22.0
CL1	Clearance of IRI from central plasma compartment	0.43	mL/hr	20.5
K_in_	Influx rate of IRI into tumor without laser	0.6050E-02	1/hr	21.1
K_out_	Efflux rate of IRI from tumor without laser	0.3500E-01	1/hr	11.1
b_1_	Light-induced influx enhancement factor for IRI	6.05	–	15.5
b_2_	Light-induced efflux enhancement factor for IRI	0.98	–	19.0
CL2	Clearance of SN-38 from central plasma compartment	0.19	mL/hr	4.07
CLm	Clearance of IRI by conversion to SN-38	0.1779E-01	mL/hr	21.0
K_in_’	Influx rate of SN-38 into tumor without laser	0.4380E-03	1/hr	26.9
K_out_’	Efflux rate of SN-38 from tumor without laser	0.1556E-01	1/hr	44.3
b_1_’	Light-induced influx enhancement factor for SN-38	7.25	–	28.8
b_2_’	Light-induced efflux enhancement factor for SN-38	0.66	–	71.9

CV = Coefficient of variation.

**Table 2:** Non-compartmental analysis of PK of IRI-PoP Liposomes.

**Parameter**	**t_1/2_**	**AUC _(0-t)_**	**AUC _(0-inf)_**	**MRT _(0-inf)_**	**CL**	**V_ss_**
Unit	hr	µg/mL*hr	µg/mL*hr	hr	mL/hr	mL
IRI	5.46	1042.85	1042.87	4.31	0.36	1.55
SN-38	8.21	45.98	46.008	8.07	–	–

MRT = Mean residence time; V_ss_ = apparent volume of distribution at steady state.

To account for increased drug uptake irradiated tumors, first-order influx and efflux rate constants K_in_ and K_out_ were used. Permeabilization enhancement factors (b_1_, b_2_) are multiplied with the influx and efflux rate constants to describe the vascular permeabilization effect caused by laser treatment. The model adequately described the IRI deposition in tumor with or without laser treatment, based upon the result from maximum likelihood method using ADAPT 5 software. The model predicted that IRI reached its highest concentration in tumor tissues ∼10 hr after laser treatment, at which time the IRI concentration would be 5.1-fold greater than in non-laser treated tumors. The estimated ratio of areas under the curve for tumor IRI concentration with/without laser treatment over time was 5.2-fold, which is close to the ratio obtained experimentally using the linear trapezoidal estimation method (7.2-fold). The model supports the conclusion that laser treatment increased the IRI influx rate b1 by 6.1-fold with negligible (0.98- fold) change in the efflux rate b2 (Table 1).

First-order elimination kinetics were used to model the PK of SN-38. In the model (Fig 8**A**) CLm represents the proportion of IRI (∼4%) that is converted to SN-38. Like the parent drug, the first-order influx and efflux terms K_in_’ and K_out_’ were used to describe the drug transfer from plasma to tumor tissue. Terms b_1_’ and b_2_’ are multiplied to Kin’ and K_out_’ to account for the effect of laser treatment. Based upon the SN-38 component of the model, the SN-38 active metabolite reached its highest concentration in tumor tissues after 27–30 h, and was ∼7.9-fold higher in laser irradiated tumors than in non-irradiated tumors. The estimated ratio of AUCs for SN-38 concentration in laser-treated vs*.* non-treated tumors was 8.6-fold, which is close to the ratio obtained experimentally by the linear trapezoidal method which is 8.2-fold. Thus, the model suggests that laser treatment increased the SN-38 influx rate b1’ by ∼7.3-fold, whereas decreased the efflux rate b_2_’ by ∼0.7-fold.

Previous research from our group reported a PK/PD study of Dox-loaded PoP liposomes administered into mice bearing PDX tumors that reported when mice with PDX tumors were treated with 4 mg/kg Dox-PoP liposomes with laser treatment (200 J/cm^2^ using a 665 nm laser), tumor influx and efflux rates increased 12- and 4-fold. The Dox AUC also increased by ∼7.4 fold [27]. Interestingly, we observed similar trends here. Notably, tumor IRI deposition increased 4-fold with laser treatment 8 hr after drug administration, and for SN-38 and SN-38 G, tumor drug deposition peaked 24 h after drug administration, both metabolites were 7-fold higher with laser treatment.

The PK of PoP was also studied using a two-compartment model (**Supporting** Fig 1). Table 3 shows the model parameters. We observed that the half-life of PoP was ∼30 h, whereas the half-life of IRI was observed ∼5.5 h, which is comparable to the half-life of ONIVYDE, reported to be 6.8 h in rats [32]. Based on the longer circulating half-life of PoP, we surmise that despite improved stability of the liposomes with ASOS, there was still liposomal leakage in blood. We observed that the half-life of the active metabolite of IRI, SN-38 was slightly longer at 8.2 h.

**Table 3:** Two-compartment model parameter estimates of PoP.

**Parameters**	**Definition**	**Estimate**	**Units**	**CV%**
CL	Clearance	0.3502E-01	mL/hr	10.5
Vp	Volume of central compartment	1.312	mL	3.67
Vt	Volume of second compartment	0.7093	mL	68.35
Q	Distribution to second compartment	0.1846E-01	mL/hr	25.44

**Fig. 9:**
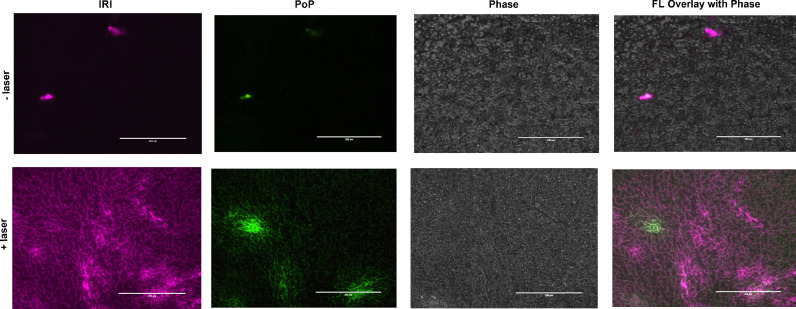
**Microdistribution of IRI and PoP in MIA PaCa-2 tumors.** Two groups of mice bearing MIA PaCa-2 tumor xenografts were administered IRI-PoP liposomes IV (15 mg/kg). One group of mice was treated with a 1 hr drug-light-interval with 665 nm laser at 200 mW/cm^2^ (250 J/cm^2^). All mice were sacrificed 8 hr after drug administration. Selected areas of tumor slices were imaged. The purple signal represents IRI distribution and PoP is shown in green. Scale bar, 200 µm. Representative images for *n* = 3 mice per group.

The spatial distribution of both IRI and PoP in tumor sections was assessed using fluorescence microscopy. Mice bearing MIA PaCa-2 tumors were administered with 15 mg/kg IRI-PoP liposomes. Mice receiving laser treatment were treated a laser fluence rate of 300 mW/cm^2^ for a total fluence of 300 J/cm^2^, with a 1 h drug-light-interval. Eight hr following drug administration, tumors were harvested, frozen in liquid nitrogen, sectioned, and imaged. As shown in Fig 9, IRI (purple) and PoP (green) could be imaged in these sections. Representative micrographs show that the tumor sections from mice treated with IRI-PoP liposomes and laser irradiation had much higher accumulation of IRI and PoP than tumor sections from mice that received no laser treatment. The distribution of IRI appeared uniform throughout the tumor.

**Fig. 10:**
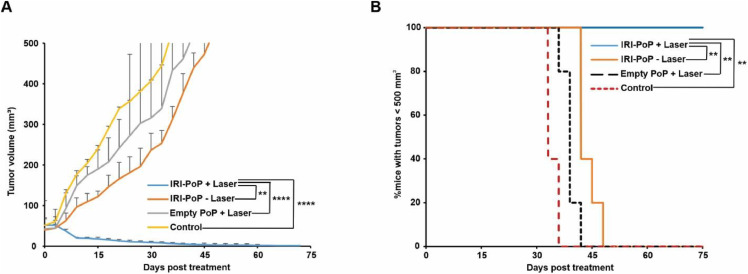
**Anti-tumor efficacy of a single treatment of IRI-PoP liposomes on low-passage PDX tumors.** Mice bearing #14,312 tumors were injected intravenously with 15 mg/kg IRI-loaded PoP liposomes or the equivalent PoP dose in empty liposomes. One hr after drug administration, mice in the +laser groups were treated with a 665 nm laser at a fluence rate of 200 mW/cm^2^ (250 J/cm^2^). Mice were given a single treatment and were sacrificed when the tumors grew to 1.5 cm in any dimension. **A).** Tumor volume progression. Each data point shows mean ± std. dev. **B)** Percentage of mice with tumors < 500 mm3 for the indicated treatments. Statistical analysis of tumor volume data on day 30 post treatment was performed using one-way ANOVA by Tukey's multiple comparison's test, and that of Kaplan Meier curve was performed by Log-rank (Mantel-Cox) test, *P < 0.05, **P < 0.01, ***P < 0.001, ****P <0.0001. *N* = 5 mice per group.

Anti-tumor efficacy of IRI-PoP liposomes also was assessed in a low-passage, patient-derived xenograft (PDX) model. This low-passage PDX model exhibits low vascularization and high cell-to-vessel distances [33] and desmoplasia of clinical tumors, and also feature low permeability and perfusion [34] that also contributes to the treatment resistance of clinical pancreatic cancer. Mice were treated when tumors reached 5–7 mm in diameter. Fig 10**A** shows that a single treatment with 15 mg/kg IRI-PoP liposomes and tumor laser irradiation resulted in complete regression of PDX tumors. Monotherapies of IRI-PoP liposomes without laser irradiation, or empty PoP liposomes with laser irradiation had minimal anti-tumor efficacy. The tumors that were treated with IRI-PoP liposomes and laser reduced to ∼half their size by the 15th day from the day of the treatment, whereas mice from other groups had grown to more than double their original size. Fig 10**B** shows that all mice that were treated with IRI-PoP liposomes and laser irradiation survived throughout the study period without tumor regrowth, whereas median survival of all other groups to the protocol tumor volume limit was not statistically different from vehicle controls.

**Fig. 11:**
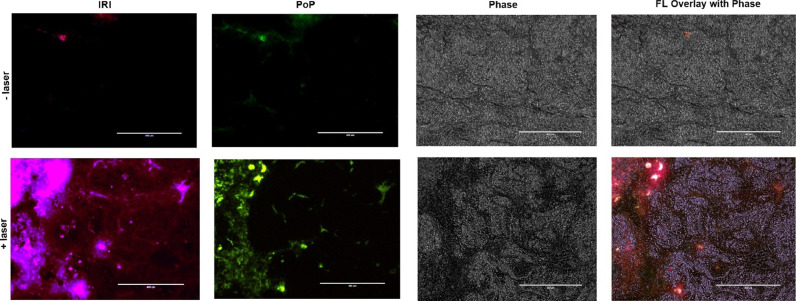
**Microdistribution of IRI and PoP deposition in PDX tumors.** Mice bearing PDX tumors were administered with IRI-PoP liposomes IV (15 mg/kg). One group of mice was treated 1 hr after liposome administration with a 665 nm laser at 200 mW/cm^2^ (250 J/cm^2^). All mice were sacrificed 8 hr after drug administration. Selected areas of tumor sections were imaged. The purple signal represents IRI distribution and PoP is false-colored green. Scale bar, 400 µm. Representative images from *n* = 3 mice per group.

IRI and PoP microdistribution in PDX tumors also was assessed. Fig 11 shows the markedly increased deposition of both IRI and PoP in irradiated tumors compared to those that were not irradiated. Compared deposition in MIA PaCa-2 tumors, the more variable tumor tissue morphology in the PDX tumors paralleled the greater heterogeneity in drug microdistribution. In general, the PoP and IRI signals had similar spatial distribution patterns in the PDX tumors. The enhancement of PoP and IRI deposition support the conclusion that the enhanced drug accumulation following laser treatment resulted from liposomes entering the tumor following vascular permeabilization by the laser treatment.

Although these results in human MIA-PaCa-2 and PDX subcutaneous tumors reveal a potent ablation mechanism, some limitations should be noted. These studies were not carried out in orthotopic tumor models, which can be a useful tool for pancreatic cancer research [35]. Furthermore, these tumor models also used immunocompromised mice, but it is known that the immune response against pancreatic cancer is important [36]. Ongoing studies are in progress to assess IRI-PoP in immunocompetent pancreatic tumor models. Another limitation of this study is the nature of the physical ablation using surface irradiation and size of the mouse tumors treated. Even though murine tumors were effectively ablated, they were well less than a centimeter. Our previous work showed that enhanced CPT drug delivery decreased with tumor depth as even red light is rapidly attenuated in tissues [37]. Unresectable advanced pancreatic tumors in humans are larger and also close to critical pancreatic vessels, so that precise light delivery using approaches such as interstitial PDT are required [38]. The PK model assumed that the effect of drug formulation and triggered release was mediated by enhanced tumor drug influx and efflux induced by the laser treatment as a consequence of phototherapy-induced vascular damage and permeabilization. However, this is a relatively simplified PK model that illustrates the experimental data, hence an elaborate PK study with more experimental data points enabling a more realistic physiologically based PK/PD model, especially with regard to IRI metabolism would ensure better understanding of the pharmacokinetic and pharmacodynamic behavior of the liposomes.

## Conclusions

PoP could be incorporated readily into liposomes that were formed from a similar lipid composition and the same trapping agent (sucrosulfate) as ONIVYDE. Compared to the AmSO_4_ trapping agent, ASOS provided PoP liposomes with improved serum stability. In our prior work with AmSO_4_ as the trapping agent, IRI-sphingomyelin-PoP liposomes were found to release >30% of the drug in adult bovine serum at 37 °C in 3 h [39]. The small amounts of PoP required to confer photosensitivity upon IRI-PoP liposomes did not interfere with the remote loading of IRI, did not disrupt the small unilamellar nature of the liposomes, and did not trigger drug leakage under physiological incubation conditions in the absence of red-light exposure. PoP mediated rapid light-triggered release of IRI upon exposure to 665 nm laser irradiation. In two different pancreatic tumor models in mice, IRI-PoP liposomes elicited effective and durable tumor responses, ablating tumors with a single treatment. Biodistribution and pharmacokinetic studies of IRI and its metabolites SN-38 and SN-38 G in tissues showed appreciably higher accumulation of all these species in tumors that received irradiation. Laser treatment increased tumor exposure to the active metabolite SN-38 by 8.2-fold; a PK model estimated the exposure as 8.6-fold, in agreement with the observed data. Taken together, these data demonstrate that a novel IRI-PoP liposome formulation promotes tumor ablation based on enhanced drug delivery. Further exploration of this liposome formulation and tumor treatment modality for pancreatic cancer applications is warranted.

## Author contributions

SG: Experimental design, carried out the experiments, interpreted data and manuscript writing. DL: Assistance in pharmacokinetic analysis. BS: Pharmacokinetic analysis. DJ: Carried out cryo electron microscopy studies. JO: Data interpretation and manuscript editing. RMS: Experimental design, data interpretation and assistance in manuscript editing. JFL: Experimental design, data interpretation and manuscript writing.

## Declaration of Competing Interests

J.F.L declares interest in POP Biotechnologies. The other authors declare that they have no known competing financial interests or personal relationships that could have appeared to influence the work reported in this paper.

## Appendix Supplementary materials

Image, application 1
