# Supplementary material for: Single-treatment tumor ablation with photodynamic liposomal irinotecan sucrosulfate
Source: Transl Oncol. 2022 Mar 12;19:101390. doi: 10.1016/j.tranon.2022.101390 (PMC8918863; doi:10.1016/j.tranon.2022.101390)
Supplement: Supplementary file 1 [file mmc1.pdf]

Supporting Information

Single-Treatment Tumor Ablation and Pharmacokinetic Analysis with Photodynamic Liposomal Irinotecan Sucrosulfate

Sanjana Ghosh, Boyang Sun, Dushyant Jahagirdar, Dandan Luo, Joaquin Ortega, Robert M. Straubinger and Jonathan F. Lovell

Table S1. Assay Performance: Calibrator Samples

Analyte	Calibrator Accuracy (%)		Calibrator Precision (%)	
	Mean	Range	Mean	Range
**IRI**	99.9	94.3-103	3.21	1.09-6.22
**SN-38**	99.9	94.8-104	4.51	2.17-7.16
**SN-38G**	100	92.9-104	5.38	1.09-7.77

Table S2. Assay Performance: Quality Control Samples

Analyte	Quality Control Accuracy (%)		Quality Control Precision (%)	
	Mean	Range	Mean	Range
**IRI**	105	99.3-110	7.41	2.50-11.3
**SN-38**	101	98.7-102	6.18	3.63-10.6
**SN-38G**	100	98.2-104	11.1	9.14-13.7

Table S3. Biodistribution of IRI and its metabolites

Analyte_Time (in hours)	Tumor (- Laser) ng/g	Tumor (+ Laser) ng/g	Plasma ng/mL	Liver ng/g	Spleen ng/g
IRI_1	2835	2559	140000	19310	34310
IRI_2	8373.33	17626.67	116866.7	20813.33	44560
IRI_8	7489	27480	47400	43820	65560
IRI_24	1564	17320	764	22040	17100
IRI_48	545.7	17320	2.8125	1651	2214
IRI_96	681.86	3773.333	2.074	103.3333	1170.667
SN-38_1	21.12	18.42	3537.1	1919	54
SN-38_2	64.8	183.7333	3050.4	1774.667	55.2
SN-38_8	56.01	249.2	2225.95	2544	55.8
SN-38_24	46.77	324.5	277.55	1201.3	40.02
SN-38_48	26.67	324.5	6.5875	222.2	8.47
SN-38_96	18	229.6	2.04	15.08	4.332
SN-38G_1	3.504	2.761	42.65	2.294667	3.722667
SN-38G_2	17.48	47.10667	73.96667	2.132	3.310667
SN-38G_8	19.87	75.76	51.6	7.82	9.051
SN-38G_24	16.08	86.7	21.025	1.669333	4.757
SN-38G_48	3.268	86.7	4.0325	1.028	0
SN-38G_96	3.313333	12.64	1.342	0	0

Each data point shows mean for n=4 mice per group.

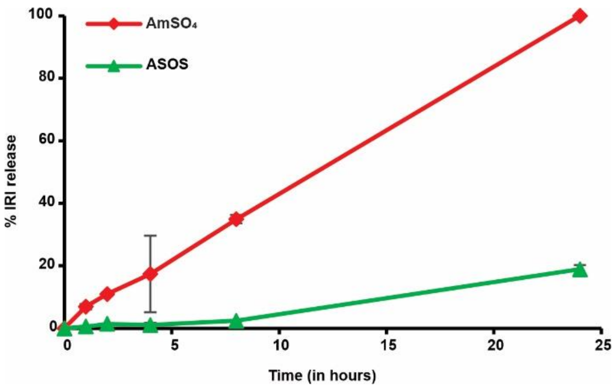

Figure S1: Effect of drug-complexing agent on in vitro serum stability of IRI loaded in 1%PoP liposomes. IRI was loaded into liposomes containing 1 mol.% PoP with different remote loading buffers. In vitro serum stability was tested by incubation in 20% bovine serum at 37 °C. Figure shows %IRI release over time. Mean ± std. dev. for n=3.

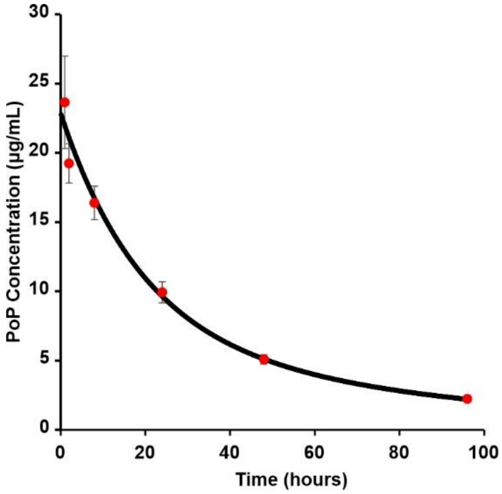

Figure S2: Pharmacokinetics of PoP administered as IRI-PoP liposomes. Mice were injected with IRI-PoP liposomes containing 15 mg/kg IRI concentration and then laser-irradiated with a total fluence of 250 J/cm^2 using a 665 nm laser with fluence rate of 200 mW/cm^2 1 hour after drug administration. The plot symbols show plasma concentrations of PoP measured by fluorescence measurements. The smooth curve represents the prediction of a two-compartment model developed describe the PK profile. Data represent mean ± std. dev. for n=4 mice per group.
